# Monocyte-to-macrophage switch reversibly impaired by Ibrutinib

**DOI:** 10.18632/oncotarget.26744

**Published:** 2019-03-08

**Authors:** Isacco Ferrarini, Antonella Rigo, Alessio Montresor, Carlo Laudanna, Fabrizio Vinante

**Affiliations:** ^1^ Department of Medicine, University of Verona, Verona, Italy; ^2^ Section of Hematology, Cancer Research & Cell Biology Laboratory, University of Verona, Verona, Italy; ^3^ Division of General Pathology, University of Verona, Verona, Italy

**Keywords:** Ibrutinib, monocyte, macrophage differentiation, fibrocyte-like cells, adhesion

## Abstract

Ibrutinib is increasingly adopted for treating lymphoid malignancies. While growing amounts of data pile up about Ibrutinib mechanism of action on neoplastic B cells, little is known about its impact on other immune cells.

Here we investigated the effect of Ibrutinib on monocyte/macrophage functions. (1) Ibrutinib treatment of purified human monocytes affected both chemoattractant-triggered inside-out as well as integrin-mediated outside-in signaling events, thus provoking defective adhesion and spreading on purified integrin ligands, respectively. (2) In *in vitro* cell-culture experiments, Ibrutinib promoted a differentiation shift of monocytes to fibrocyte-like cells, characterized by the acquisition of a typical elongated cell morphology. Importantly, this clear-cut shape transition also occurred upon culturing monocytes with sera derived from Ibrutinib-treated patients, thus clearly suggesting that the drug concentrations achievable *in vivo* can generate the phenotypic shift. (3) Ibrutinib-induced fibrocyte-like cells showed adhesion deficiency, altered phagocytic properties, and, with respect to macrophages, they acquired the capability of generating larger amounts of reactive oxygen species, possibly displaying different metabolic activities.

Taken together, our results indicate that Ibrutinib has profound effects on the monocyte/macrophage immunobiology. They may finally shed some light about the biological ground of several Ibrutinib-related toxicities.

## INTRODUCTION

Ibrutinib, a first-in-class Bruton's tyrosine kinase (BTK) irreversible inhibitor, also targeting other kinases such as ITK [1] and EGFR [2], is changing treatment paradigms of some chronic lymphoproliferative disorders such as chronic lymphocytic leukemia (CLL), mantle cell lymphoma and Waldenström macroglobulinemia [3]. The Ibrutinib-main targeted enzyme BTK is a cytoplasmic nonreceptor tyrosin kinase, belonging to the TEC (tyrosine kinase expressed in hepatocellular carcinoma) kinase family, which transmits and amplifies signals downstream from a wide variety of molecules including G-protein coupled receptors, antigen receptors and integrins [4]. Beyond its fundamental role in B-cell ontogenesis, BTK functions have been dissected in detail in the context of normal and neoplastic mature B lymphocytes, where it is mostly involved in signal transduction from chemokine receptors and B-cell receptor (BCR), leading to the acquisition of the high-affinity binding conformation of integrins and to PI3K-AKT and NF-kB pathway activation, respectively. These effects ultimately regulate cell migration and homing, as well as cell survival, expression of activation markers and antibody production [4–6]. However, expression of BTK and other kinases targeted by Ibrutinib is not restricted to the B cell compartment. Also T lymphocytes [1], NK cells [7], neutrophils [8] and monocyte-macrophages [9] express at least some of them, thus suggesting that Ibrutinib effect is not restricted to B lymphocytes, as previously considered, but rather may have a wider modulatory effect on innate and adaptive immune responses. For instance, in neoplastic disease models it can subvert Th2 immunity by skewing CD4^+^ T cell populations toward a Th1 profile [1], improving responses of both checkpoint inhibitors [10] and chimeric antigen receptor T-cells [11] against lymphoid cancers. On the other side, Ibrutinib treatment may dampen TREM-1-mediated activities in human neutrophils, impairing substantial aspects of first-line defense against pathogens such as adhesion, oxidative burst and degranulation [12]. These findings, albeit inconclusive so far, have translational relevance if we consider both the biological ground on which Ibrutinib must act, namely neoplastic tissues with essential and reciprocal interactions between leukemic and bystander inflammatory cells, and the immunological profile of treated patients, often heavily compromised. Notably, it is becoming apparent from recent clinical reports that Ibrutinib may predispose to life-threatening opportunistic infections not otherwise expected in CLL patients, suggesting the immune dysregulation triggered by Ibrutinib might have substantial implications in clinical practice [13].

Monocytes are key components of innate immunity. They derive from bone marrow myeloid progenitor cells, circulate in peripheral blood for a short time and, under inflammatory conditions, adhere to the endothelial barrier and transmigrate to peripheral tissues, where they can differentiate into divergent cell populations such as macrophages, monocytoid dendritic cells or fibrocytes, depending on the microenvironment stimuli they encounter [14, 15]. Each of these cell types can acquire further polarization profiles, within a continuous spectrum of functional states, contributing to delineate the immunostimulatory/anti-neoplastic or immunotolerant/pro-neoplastic properties of the microenvironment [16]. Since Ibrutinib has become available for biological and clinical uses, just a few studies have assessed its impact on monocyte-macrophages pathophysiology. Fiorcari and colleagues have demonstrated that Ibrutinib slightly modifies the monocytic population in treated CLL patients, showing a higher positivity for CD206 and Tie2 receptor. Furthermore, it induces the expression of the immunosuppressive receptors CD163 and PD-L1 as well as the secretion of IL-10 by nurse-like cells, shifting their polarization towards an immune tolerant M2 phenotype [17]. Whether this could be really crucial for CLL microenvironment remains to be established. In fact, histopathological evaluation of bone marrow specimens from Ibrutinib-treated patients has demonstrated the *in vivo* disruption of interactions between macrophages and neoplastic cells, being deprived of important pro-survival stimuli [18]. However, dysfunctional cell-cell interplay triggered by Ibrutinib may also reduce FcγR-mediated phagocytosis and impair co-treatments with monoclonal antibodies [19]. Even pro-inflammatory cytokines production triggered by FcγR is decreased in Ibrutinib-treated monocytes, potentially blocking natural and therapy-induced antineoplastic mechanisms [20].

Overall, knowledge about the consequences of Ibrutinib treatment on mononuclear phagocytes is still scanty and contradictory, with results often limited to specific disease settings. This prompted us to comprehensively investigate the effects of Ibrutinib on more general aspects of monocyte-macrophage pathophysiology. We demonstrate that Ibrutinib inhibits rapid integrin-mediated adhesion of healthy human monocytes and switches their differentiation fate towards very elongated fibrocyte-like cells, characterized by a peculiar pattern of effector functions. This may expand our insight into the biologic bases of Ibrutinib efficacy and help us better understand the mechanisms underlying some treatment-related toxicities.

## RESULTS

### Ibrutinib inhibits rapid beta2-integrin-mediated adhesion and spreading of monocytes

**Figure 1:**
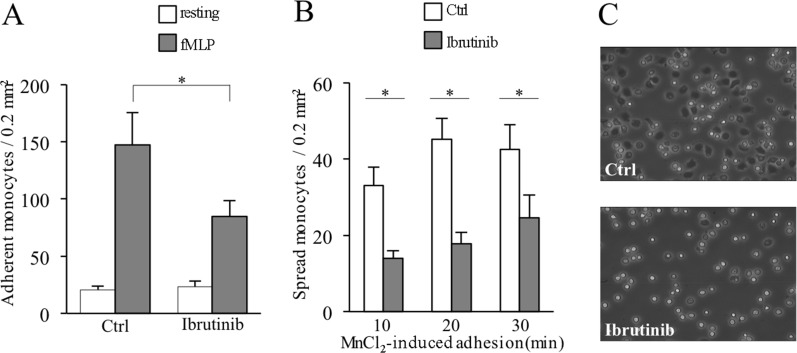
Ibrutinib-induced inhibition of inside-out and outside-in signaling of LFA-1 in monocytes Shown are control cells (Ctrl) versus Ibrutinib-treated cells (Ibrutinib). (**A**) Number of monocytes adherent to ICAM-1 upon triggering with buffer (resting) or 25 nM fMLP. (**B**) Number of monocytes spread on ICAM-1 upon triggering with 1 mM MnCl_2_ for 10, 20, 30 minutes. Data presented are mean ± SD and are representative of 8 experiments. ^*^p < 0.001 by one-way analysis of variance and Bonferroni *post-hoc* analysis. (**C**) Representative image of spread monocytes on ICAM-1 upon triggering with 1 mM MnCl_2_ for 10 minutes. Images are captured by contrast phase microscope (10x) and are representative of 3 experiments.

To evaluate how Ibrutinib may affect monocytes recruitment, we first analyzed the capability of the chemotactic factor *N*-formylmethionyl-leucyl-phenilalanine (fMLP) to elicit integrin-mediated rapid adhesion in healthy purified peripheral blood untouched monocytes (negative selection) treated or not with Ibrutinib. We observed a significant reduction of the capability of Ibrutinib-treated monocytes to adhere to immobilized ICAM-1, ligand for the beta-2 integrins LFA-1 (CD11a/CD18) and Mac1 (CD11b/CD18), suggesting that Ibrutinib inhibits chemoattractant-triggered inside-out signaling leading to rapid beta-2 integrin activation in monocytes (Figure 1A).

We next treated monocytes with MnCl_2_ to artificially induce a beta-2 integrin conformational state corresponding to increased affinity independently of inside-out signaling, and evaluated the capability of adherent cells to spread. Although cells were able to adhere, as expected, we observed an important reduction of spread cells in Ibrutinib-treated monocytes with respect to control cells, suggesting that Ibrutinib may also inhibit integrin-elicited outside-in signaling (Figure 1B–1C). Thus, Ibrutinib impairs bi-directional signaling related to beta-2 integrin activity, dampening monocyte firm adhesion and spreading.

### Ibrutinib impairs monocyte-to-macrophage differentiation *in vitro*: morphology and flow cytometry

**Figure 2:**
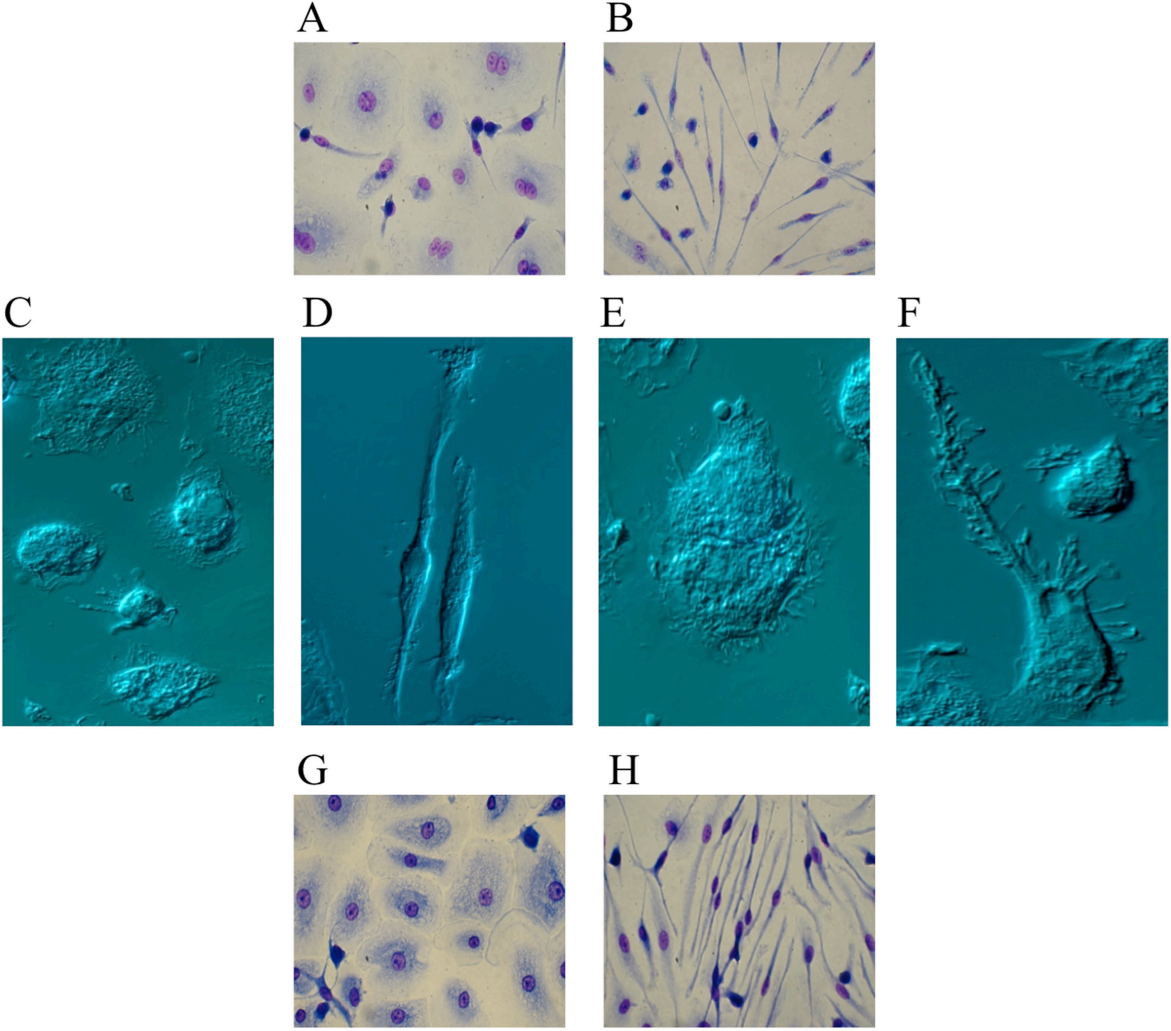
Ibrutinib-induced differentiation into fibrocyte-like cells Purified human monocytes were cultured in the presence of 100 ng/mL M-CSF. (**A–F**) Monocytes were cultured in serum-containing medium in the absence (A, C, E) or presence (B, D, F) of 10 μM Ibrutinib. After 10 days, cells were air-dried, fixed and stained with May-Grunwald and Giemsa solutions for optic microscopy (A, B) or captured unstained for interference microscopy (C–F). Monocytes cultured with only M-CSF (A, C, E) displayed a fried-egg or slightly spindle shape with round nuclei, typical of M-CSF-differentiated cells. Monocytes cultured with M-CSF + Ibrutinib (B, D, F) acquired a very elongated morphology with oval nuclei, consistent with a clear-cut differentiation into fibrocyte-like cells. Images captured by interference microscopy at higher magnification (100x) showed the morphological properties of a monocyte cultured with only M-CSF (E) or undergoing fibrocyte differentiation upon stimulation with M-CSF + Ibrutinib (F). In the latter case, numerous filopodia extend beyond the leading edge of the cytoplasmic projection. Images are representative of 10 separate experiments. (**G–H**) Monocytes were cultured with 40% serum of a CLL patient withdrawn before (G) and after (H) the beginning of Ibrutinib treatment. After 10 days, cells were air-dried, fixed and stained with May-Grunwald and Giemsa solutions. A representative experiment out of 6 is shown.

To assess whether Ibrutinib may affect monocyte-to-macrophage differentiation, we cultured healthy purified peripheral blood monocytes for 10 days in medium containing macrophage colony-stimulating factor (M-CSF), a major promoter of macrophage differentiation [21], in the absence or presence of Ibrutinib. Cells cultured with only M-CSF displayed a spread, fried-egg, shape typical of macrophages or, to a lesser extent, a slightly spindle morphology [22, 23]. Instead, in Ibrutinib-treated monocytes we observed the acquisition of evident morphological changes as soon as 5 days, which progressively accentuated over the next few days. Monocytes acquired a very elongated shape with a central oval nucleus, consistent with a dramatic differentiation into fibrocyte-like cells (Ibrutinib-induced fibrocyte-like cells, IIF) (Figure 2A–2D). Although attempts to better identify them by immunophenotypical methods have been made by us and others, specific, reliable and standardized markers to distinguish fibrocytes from macrophages and fibroblasts are lacking and morphology along with haematopoietic origin are the main characteristics we should consider [24]. Flow cytometry analysis confirms that M-CSF-differentiated macrophages (Mϕ) and IIF are phenotypically related, as they share most surface molecules belonging to the monocyte-macrophage lineage, with a prevalent expression of the anti-inflammatory/M2 markers (CD206, CD163 and CD200R). Only HLA-DR reached statistical significance, as it was found consistently reduced in IIF, possibly resulting in poorer antigen presentation ability (Supplementary Table 1).

IIF tried to self-organize by aligning together along the same direction, just as it happens for most elongated cells. Furthermore, we observed the emergence of numerous, filopodia-like, protrusions extending beyond the leading edge of cytoplasmic projections, suggesting a consistent cytoskeletal rearrangement (Figure 2E–2F).

We observed that the acquisition of spindle shape also occurred upon addition of Ibrutinib to terminally M-CSF-differentiated macrophages (Mϕ), possibly suggesting that Ibrutinib could affect the differentiation program of tissue resident macrophages as well. Conversely, if Ibrutinib-containing medium was replaced by Ibrutinib-free medium, IIF lost their typical elongated morphology taking on a more rounded appearance, consistent with macrophage transdifferentiation (data not shown). Therefore, Ibrutinib effects on monocyte-macrophage differentiation are fully reversible *in vitro*.

### Culture of healthy monocytes with sera of Ibrutinib-treated patients promotes fibrocyte-like differentiation

We next evaluated if the same morphological changes observed upon Ibrutinib *in vitro* treatment could be also obtained at concentrations of drug achievable *in vivo*. Thus, we cultured purified healthy monocytes with sera derived from six patients affected by CLL, collected either before the beginning of Ibrutinib treatment or after 1 month of continuous daily therapy at the dose of 420 mg/day. We found that, in three out of six cases, monocytes cultured for 10 days with post-treatment serum showed a dramatic differentiation into fibrocyte-like cells compared to those cultured with pre-treatment serum (Figure 2G–2H), suggesting that Ibrutinib serum concentrations achievable in patients treated with the drug at 420 mg/die are potentially able to induce a consistent shift in the differentiation route of resident monocytes. The other three cases showed a less evident fibrocyte-like differentiation, with co-presence of spread-shaped and elongated-shaped cells in the same culture (not shown). This variability may be the result of either different time intercurring between Ibrutinib intake and serum withdrawal, or possible interactions between Ibrutinib and other concurrent treatments, determining variability in plasma peak concentrations.

### Ibrutinib may exert a serum-contrasting effect on monocyte differentiation

**Figure 3:**
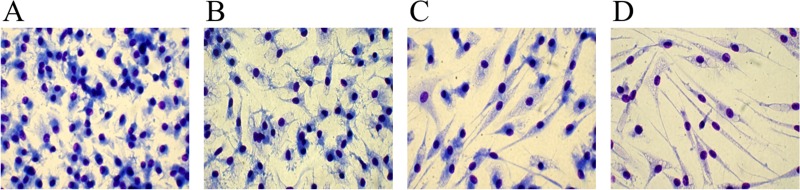
Differences between serum-containing and serum-free media in fibrocyte-like cells differentiation Purified human monocytes were cultured in Neurobasal medium in the presence of 100 ng/mL M-CSF. (**A, C**) 20% FCS was added to the medium and cells were cultured in the absence (A) or presence (C) of 10 μM Ibrutinib. (**B, D**) Cells were cultured without adding serum, in the absence (B) or presence (D) of 10 μM Ibrutinib. A representative experiment out of 3 is shown. Images are captured at 40x magnification.

Despite the use of serum containing medium, which is known to counteract fibrocyte differentiation due to the presence of serum amyloid P component (SAP) [25, 26], Ibrutinib shifts monocytes towards a fibrocyte morphology. To better investigate whether Ibrutinib could affect the acquisition of the typical rounded macrophage phenotype triggered by serum, we needed to remove serum from culture, though maintaining an adequate number of viable cells. To do that, we tested several serum-free (and cytokine-free) media and finally we choose Neurobasal medium for subsequent experiments. Monocytes cultured for 10 days with serum-supplemented Neurobasal medium acquired the rounded macrophage morphology (Figure 3A). This partially turned into a more elongated shape after culturing cells in serum-free Neurobasal medium (Figure 3B). In the presence of Ibrutinib, the typical fibrocytic differentiation occurred and was greatly enhanced in the serum-free condition (Figure 3C–3D). This suggests that Ibrutinib may dampen the effects of serum components on monocyte-to-macrophage differentiation.

### Pentraxin 3 release in macrophages and Ibrutinib-induced fibrocyte-like cells

**Figure 4:**
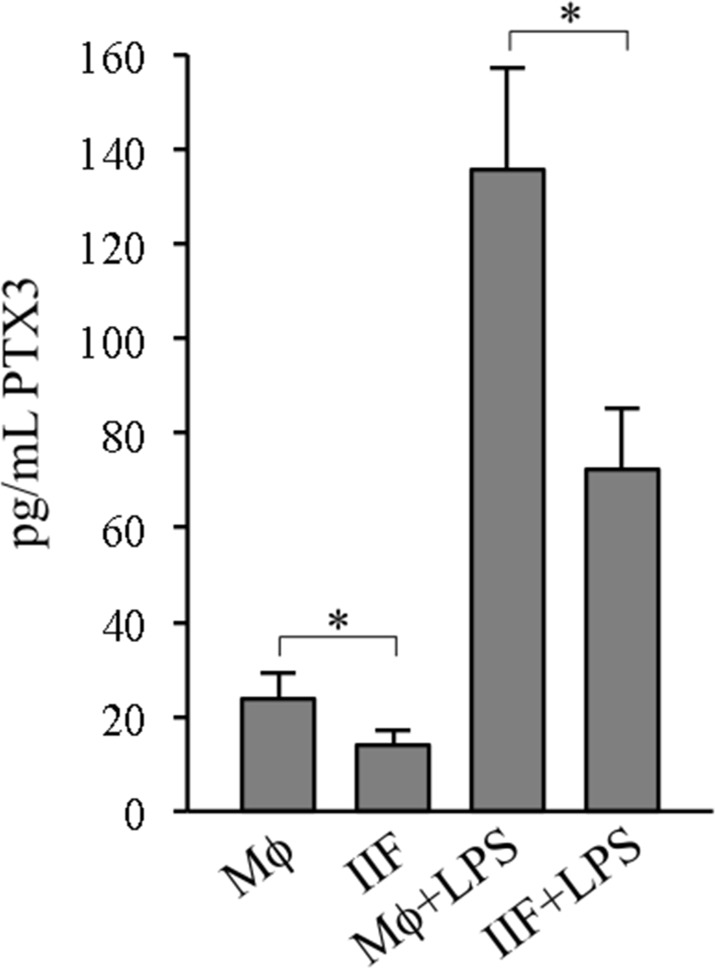
PTX3 release in differentiated cells SN derived from cells cultured for 10 days were collected and analysed for PTX3 by ELISA, either in basal conditions (Mϕ, IIF) or after 24 hour stimulation with 1 ng/mL LPS (Mϕ+LPS, IIF+LPS). Data presented are mean ± SD and are representative of 6 experiments. ^*^p < 0.05.

Pentraxin 3 (PTX3) is produced, under appropriate conditions, by monocyte-macrophages, and it has been recognized as a major promoter of fibrocyte differentiation, both *in vitro* and in murine models of pulmonary fibrosis [27]. Thus, we wondered whether PTX3 production could be correlated to IIF differentiation. Supernatants (SN) collected from Mϕ or IIF cultures were analyzed for PTX3 levels by ELISA. Unexpectedly, PTX3 concentrations were significantly lower in supernatants collected from IIF compared to those collected from Mϕ, suggesting that Ibrutinib negatively affects PTX3 expression and/or release, thus supporting a negative correlation between PTX3 production and differentiation to fibrocyte-like cells induced by Ibrutinib (Figure 4). Even after lipopolysaccharide (LPS) stimulation, known to enhance PTX3 synthesis [28], IIF released a significantly lower amount of PTX3 with respect to Mϕ (Figure 4), showing that Ibrutinib may impair monocyte-to-macrophage differentiation independently of PTX3 pathway.

### Ibrutinib-induced fibrocyte-like cells show adhesion deficiency

**Figure 5:**
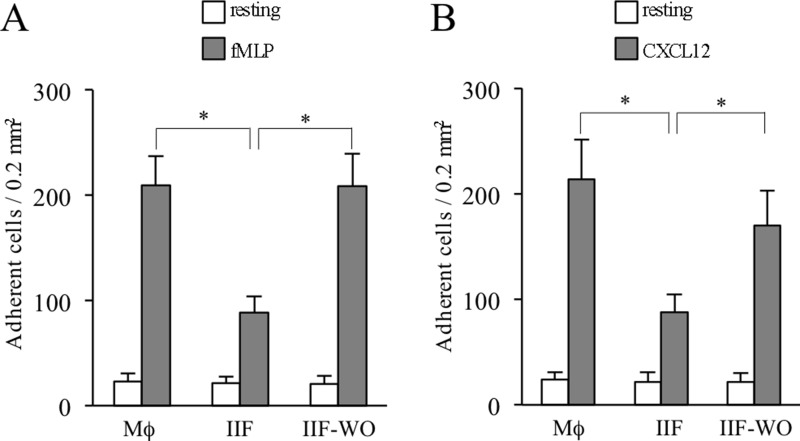
Ibrutinib-induced adhesion impairment in differentiated cells Data are presented for Mϕ, IIF and IIF after washing out Ibrutinib (IIF-WO). (**A**) Number of cells adherent to ICAM-1 upon stimulation with buffer (resting) or fMLP. (**B**) Number of cells adherent to ICAM-1 upon stimulation with buffer or CXCL12. Data presented are mean ± SD and are representative of 3 experiments. ^*^p < 0.001 by one-way analysis of variance and Bonferroni *post-hoc* analysis.

Having identified a phenotypic shift of monocytes under Ibrutinib treatment and since nothing is known about functional characteristics of IIF, we next performed a series of experiments in order to characterize their adhesion ability, phagocytic properties and metabolic activity. To investigate whether Mϕ and IIF may display different adhesion capability, we performed static adhesion assays to beta2-integrin ligands in terminally differentiated cells. We found that IIF had a significant adhesion deficiency compared to Mϕ upon stimulation with fMLP or CXCL12, a CXC chemokine constitutively expressed in bone marrow microenvironment. Importantly, after removal of Ibrutinib from culture medium for 48 hours, the adhesion capability of IIF became almost identical to that of Mϕ, probably due to neosynthesis of targeted enzymes (Figure 5A–5B). These findings show that IIF display a defective integrin-mediated adhesion; this may possibly affect the capability of establishing stable interactions with surrounding adjacent cells, thus disturbing microenvironment integrity.

### Bacterial and fungal phagocytosis in macrophages and Ibrutinib-induced fibrocyte-like cells

**Figure 6:**
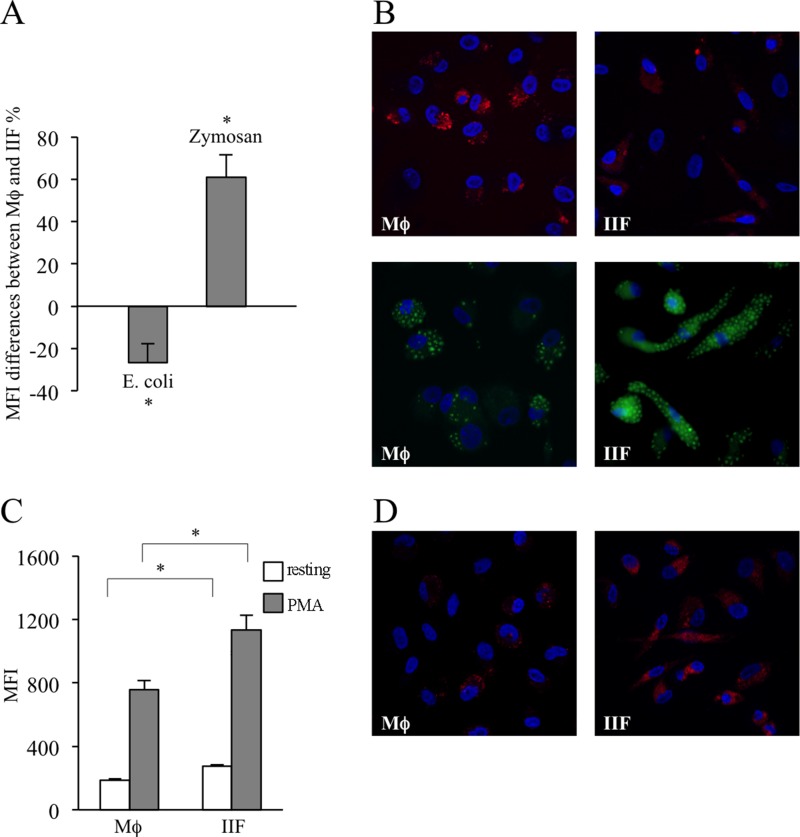
Evaluation of phagocytic ability and ROS production in differentiated cells The results have been evaluated in flow-cytometry and expressed as MFI and by fluorescence microscopy. They are mean ± SD and are representative of 3 experiments. (**A**) Mϕ and IIF were exposed to *E. coli* particles or zymosan labelled with a pH-sensitive fluorophore. MFI differences between macrophages and fibrocytes, expressed as percentages, are shown. In each condition autofluorescence was subtracted. ^*^p < 0.05. (**B**) Fluorescence microscopy of Mϕ (upper left and lower left) and IIF (upper right and lower right) incubated with *E. coli* particles (upper panels) or zymosan (lower panels). (**C**) Mϕ and IIF were incubated with CellRox Deep Red reagent, both in basal condition (resting) and after stimulation with PMA. In each condition autofluorescence was subtracted. ^*^p < 0.001. (**D**) Fluorescence microscopy of resting Mϕ (left panel) and IIF (right panel) incubated with CellRox Deep Red reagent.

Phagocytosis is a tightly regulated process, which involves different receptors and intracellular pathways depending on the specific target to be internalized. Bacterial phagocytosis mainly involves Toll-like receptor (TLR) 4 and TLR9, stimulated by LPS and hypomethylated CpG DNA motifs, respectively [29, 30], whereas fungal internalization relies on dectin-1, TLR2 and TLR6, mediating β-glucans recognition [31, 32]. To investigate whether Ibrutinib could interfere with bacterial and fungal phagocytic activity, we exposed Mϕ and IIF to fluorescently labelled *E. coli* particles and fungal-derived zymosan. Very interestingly, we found that, with respect to Mϕ, *E. coli* phagocytosis was inhibited in IIF. Conversely, zymosan phagocytosis was significantly increased. This highlights the emergence of an important dichotomy in signaling pathways regulating bacterial versus fungal-specific phagocytosis, oppositely affected by Ibrutinib (Figure 6A–6B, Supplementary Figure 1).

### Ibrutinib-induced fibrocyte-like cells produce larger amounts of superoxide anion

Oxidative burst is a peculiar function of professional phagocytes, dependent of NADPH oxidase activation, and devoted to oxygen free radicals (ROS) production leading to amplification of antimicrobial and antineoplastic response without affecting the viability of ROS generating cells. We incubated Mϕ and IIF with a fluorophore sensitive to O_2_^−^ and observed that IIF produced larger amounts of ROS than Mϕ, both in basal conditions and after stimulation with phorbol 12-myristate 13-acetate (PMA), a potent NADPH oxidase inducer (Figure 6C–6D, Supplementary Figure 1). These data are consistent with a previous study demonstrating higher production of ROS in BTK-deficient neutrophils [8].

### Ibrutinib-induced fibrocyte-like cells may display a different metabolic activity compared to macrophages

**Figure 7:**
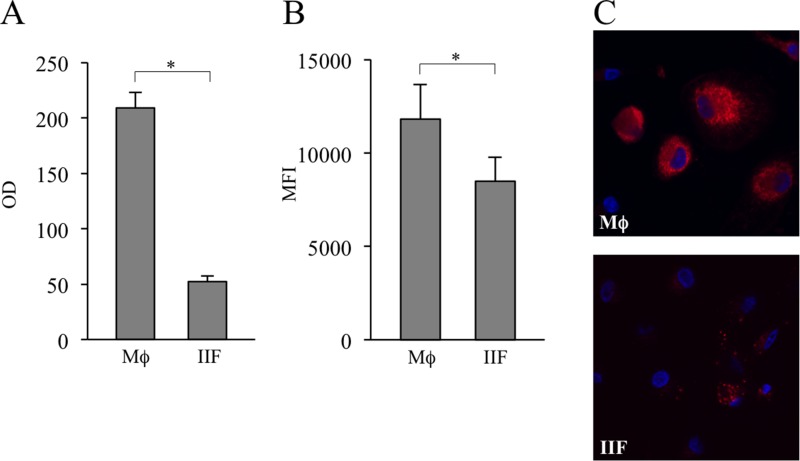
Evaluation of MTT metabolism and mitochondrial amount in differentiated cells (**A**) Mϕ and IIF were added with MTT and its catalyzation to MTT-formazan was measured. The results are expressed as optical density (OD). They are mean ± SD and are representative of 5 experiments. ^*^p < 0.001. (**B**) Mϕ and IIF were incubated with MitoTracker Deep Red FM. Then they were detached and recorded by flow cytometry. Data are expressed as MFI. In each condition autofluorescence was subtracted. The results are mean ± SD and are representative of 3 experiments. ^*^p < 0.05. (**C**) Fluorescence microscopy of Mϕ (upper panel) and IIF (lower panel) incubated with MitoTracker Deep Red FM.

Next, we wondered if IIF could rely on a different metabolic program to maintain their energetic homeostasis. The 3-(4,5-dimethilthiazol-2-yl)-2,5-diphenyltetrazolium bromide (MTT) assay, based on the enzymatic reduction of MTT to MTT-formazan catalyzed by succinate dehydrogenase, is dependent on mitochondrial respiration and indirectly assesses the cellular energy capacity of cultured cells [33]. We observed a clear-cut reduction of MTT to MTT-formazan conversion in IIF as compared to Mϕ. For the viability of cells was not affected by Ibrutinib, the effect we observed could be due to a reduced number of cells and/or decrease of the overall mitochondrial mass and/or inhibition of succinate dehydrogenase (Figure 7A). Since incubation of differentiated cells with the ΔΨm-insensitive MitoTracker probe showed decreased fluorescence intensity in IIF (Figure 7B–7C, Supplementary Figure 1), we suggest for these cells at least a reduced mitochondrial amount or a topological modification of the inner mitochondrial membrane [34].

## DISCUSSION

Among peripheral blood cells, monocytes display the unique property to differentiate, upon migration into tissues, to phenotypically and functionally different cells. This process is carefully regulated and several molecules, mainly chemokines, integrins, pentraxins and specific growth factors cooperate to select and drive distinct, though reversible, differentiation programs. Our findings suggest that Ibrutinib may interfere with the key points of monocyte-macrophage pathophysiology. The data can be summarized as follows: *a)* Ibrutinib inhibited beta-2 integrin-mediated adhesion and spreading of human monocytes; *b)* Ibrutinib affected monocyte differentiation program leading to the acquisition of fibrocytic morphology. The extent of the morphological shift depended on the presence or absence of serum in culture media; *c)* though reversible, monocyte-to-IIF differentiation involved deep changes in several mononuclear phagocyte-specific activities, as it affected in an opposite manner bacterial and fungal phagocytosis, it reduced basal as well as LPS-induced PTX3 release and promoted superoxide anion production; *d)* IIF showed chemoattractant-triggered adhesion deficiency; *e)* IIF had an impaired MTT metabolism and a lower mitochondrial amount, possibly relying on a different metabolic program.

Monocyte-to-macrophage differentiation passes through two layers of regulation, which retrace the evolutionary route of these cells. A more ancient one, orchestrated by SAP and other components of the pentraxin family acting on Fcγ immunoreceptors [27, 35, 36], and a later one, based on tyrosine kinase receptors for critical growth factors, among which M-CSF appears pivotal [22, 37]. Our findings in serum-supplemented cultures, containing both SAP and M-CSF, demonstrated that Ibrutinib heavily impairs the differentiation of human monocytes towards macrophages, rather promoting the acquisition of a very elongated morphology with a central oval nucleus, consistent with fibrocyte-like cells. As SAP and M-CSF are both considered enhancers of macrophage differentiation, Ibrutinib may inhibit signalling pathways downstream from their receptors, allowing for the phenotypic shift. Accordingly, BTK and TEC have been recognized to transduce signals downstream from FcγRs and M-CSF receptor [20, 38].

Even if large amounts of data are piling up about the ontogenesis and the morphological features of fibrocytes [39–42], their intimate biological significance remains largely obscure. This is partly due to the different cytokine cocktails that have been used to differentiate them *in vitro* and the lack of reliable surface markers to detect them *in vitro* and *ex vivo*, so that morphology still plays a major role in their definition [40–42]. Since each fibrocyte-inducing cytokine may affect intracellular signaling network, and the resulting transcriptome, in a peculiar way, it is inadequate to look at these cells as a homogeneous population. We would rather consider them as a spectrum of distinct functional states working on a common differentiation ground. Thus, we did not expect that IIF were similar to fibrocytes of different origin. Indeed, beyond affecting the differentiation thrusts, Ibrutinib generated a number of specific cell responses. First, it induced a clear-cut defect in chemoattractant-triggered beta2-integrin inside-out signaling and in integrin-elicited outside-in signaling, both in freshly isolated monocytes and in IIF. This can impair monocyte recruitment towards inflamed tissues and destabilize cell-to-matrix and cell-to-cell communications, finally perturbing the antigen-presenting functions of these cells. Notably, a bi-directional crosstalk between FcγRs and beta-2 integrins does exist [43], enabling the environmental sensing to cooperate with pentraxins and M-CSF in driving distinct differentiation routes. Then, Ibrutinib reduced the release of PTX3, the prototype of long pentraxins, involved in resistance against selected microbes (among which *Aspergillus fumigatus*), modulation of complement activity and matrix remodeling [44, 45]. The inhibition of PTX3 release became even more evident after stimulation with LPS, which is known to promote PTX3 synthesis [28]. Such a finding is consistent with previous works demonstrating that Ibrutinib-targeted kinases are involved in transducing signals downstream from the LPS receptor TLR4 [46, 47]. Accordingly, LPS-stimulated microglial cells have been reported to produce lower levels of pro-inflammatory cytokines upon Ibrutinib treatment, through the inhibition of AKT/STAT3 signaling pathways [48]. Finally, Ibrutinib affected the phagocytic capability of *E. coli* and yeast-derived zymosan particles, albeit in opposite directions. Phagocytosis of *E. coli* was impaired, whereas the one of zymosan was enhanced. This could be the result of divergent phagocytosis mechanisms selectively mediating the internalization of different microbial species. Indeed, gram-negative bacteria phagocytosis is triggered upon binding of LPS to TLR4 [49]. Instead, zymosan particles, derived from *Saccharomyces cerevisiae* cell wall and mainly composed of β-glucans, are internalized upon binding to dectin-1, TLR2 and TLR6 [50]. Although the mechanistic details remain elusive, it is likely that Ibrutinib favors some of these specific phagocytic pathways or indirectly promotes the activation of distinct small GTPases involved in cytoskeletal rearrangement necessary for the phagocytic cup formation [51].

Oxidative burst is another major property of monocyte-macrophages. The increased superoxide anion production that we observed in IIF suggests that Ibrutinib could positively regulate NADPH oxidase complex, the major source of ROS in mononuclear phagocytes. Furthermore, our data supported a wider diversity in the overall energetic cell metabolism. The reduction of MTT to MTT-formazan conversion that we pointed out in IIF could be explained by their reduced global mitochondrial mass with respect to Mϕ. This suggests that IIF may rely on a different metabolic program to provide their energy as compared to Mϕ, favoring glycolytic pathways over oxidative phosphorylation.

For the phenotypic shift also occurred by culturing monocytes with sera from Ibrutinib-treated patients, our study should help disclose a rationale for some observed Ibrutinib-related toxicities. Impaired adhesion and spreading in monocytes and IIF, as well as the decreased PTX3 release, might partly represent the basis of atypical and sometimes severe infectious complications emerging with the broad use of Ibrutinib in clinical setting. Invasive pulmonary and cerebral aspergillosis [52, 53], invasive fusariosis [54], mucormycosis [55] and pneumocystis jiroveci pneumonia [56] have been recently reported, even if the drug is taken as monotherapy and in patients not heavily pre-treated. Such complications are normally not expected in these patients, for a progressive restoration of humoral immunity usually occurs during Ibrutinib treatment [56, 57]. Particularly, and importantly, in patients taking Ibrutinib and steroids the adhesion deficiency may associate with glucocorticoid-induced M2-polarization, exacerbating the immune defect. A higher than expected incidence of cerebral aspergillosis has been actually reported in primary central nervous system lymphoma patients treated with Ibrutinib plus glucocorticoids [53].

Altogether, our results describe the impact of Ibrutinib on multiple aspects of monocyte-macrophage pathophysiology and identify a previously unrecognized phenotypic shift of possible biological and clinical interest. Most Ibrutinib-triggered phenotypic and functional changes point to decreased or altered responsiveness towards extracellular cues, either endogenous chemokines or microbial products. From chief orchestrators of innate and acquired immunity [58], mononuclear phagocytes appear to be partially downstaged under Ibrutinib treatment. Thus, IIF are likely to represent a phagocyte cell population in which the macrophage effector program has not been successfully developed. As M-CSF-induced differentiation program relies on the integrity of BTK, TEC and other proximal kinases possibly targeted by Ibrutinib [9], mechanistic investigations are ongoing to address its inhibitory effect on signaling pathways downstream from M-CSF/M-CSF-R. Moreover, given the lack of specific surface markers, gene expression profile-based studies are advisable to better characterize properties of IIF and highlight similarities and differences with other monocyte-derived cell subsets.

## MATERIALS AND METHODS

### Ethical requirements

Serum samples from CLL patients were obtained in the context of the project 1828/2010; patients diagnosed with CLL according to the current guidelines [59] participated in this study. Blood samples from healthy volunteers were obtained in the context of the project 5626/2012. Both projects were approved by the ethics committee of the Verona University Hospital. A written informed consent was obtained according to law.

### Cell cultures

Highly purified human peripheral blood monocytes (98% CD14^+^) were isolated from the buffy coats of blood samples from healthy volunteers by using Pan Monocyte Isolation kit (negative selection, Miltenyi Biotec, Bergisch Gladbach, Germany), according to the manufacturer's instructions. Purified monocytes were seeded at a density of 25 × 10^4^/mL in RPMI-1640 medium supplemented with 20% heat-inactivated fetal calf serum (FCS), 50 U/mL penicillin, 50 U/L streptomycin (Invitrogen, Carlsbad, CA, USA) and 100 ng/mL M-CSF (Peprotech, London, UK). In selected experiments, after 48 hours RPMI-1640 medium was replaced by Neurobasal medium (Invitrogen) supplemented with 100 ng/mL M-CSF, in the presence or absence of 20% FCS. Cells were treated with vehicle (dimethyl sulfoxide, DMSO, Sigma-Aldrich, St. Louis, MO, USA) or 10 μM Ibrutinib (SelleckChem, Munich, Germany) dissolved in DMSO and stored as stock solution of 10 mM. Ibrutinib dose was chosen after having performed preliminary experiments, wherein we analysed monocyte-macrophage viability by Trypan Blue exclusion assay after 72 hours M-CSF-containing culture, in the absence or presence of 0.1, 1, 5, 10 and 20 μM Ibrutinib. We choose 10 μM for subsequent experiments because it was the highest concentration not affecting cell viability.

In six experiments FCS was replaced by 40% heat-inactivated sera of CLL patients, withdrawn before the beginning of Ibrutinib treatment and after 1 month of therapy at the dose of 420 mg/day. After ten days of culture the adherent cells were washed with PBS and stained with May-Grunwald and Giemsa solutions for microscope examination. Images were also captured on interference microscope (Zeiss AxioImager 2, equipped with 6 MP Zeiss colour camera 506 and Hamamatsu 4 MP BW camera; images were acquired at 63x and 100x magnification, numeric aperture 1.46, with Zeiss Zen 2.3 acquisition and analysis software).

### Static adhesion assays

Adhesion assays were performed on 12-well glass slides coated with ICAM-1 (1 μg/mL in PBS). For inside-out signaling studies, monocytes were suspended in standard adhesion buffer (PBS + CaCl_2_ 1 mM + MgCl_2_ 1 mM + FBS 10%) at 5 × 10^6^/mL. A 20-μL cell suspension treated or not with 10 μM Ibrutinib for 1 hour was added to the wells and stimulated for 1 minute at 37°C with 5 μL fMLP (25 nM final concentration) or with 5 μL CXCL12 at 200 nM final concentration. After washing, adherent cells were fixed in ice-cold 1.5% glutaraldehyde in PBS.

For outside-in signaling studies, monocytes were suspended in PBS + 1 mM EDTA for 10 minutes. Cells treated or not with 10 μM Ibrutinib for 1 hour at 37°C were seeded on glass slides in the presence of manganese for 10, 20 and 30 minutes. Still images of adherent or spread cells in 0.2-mm^2^ fields were acquired at 320x phase-contrast magnification (numerical aperture, 0.40) with a charge-coupled device camera (ICD-42B; Ikegami, Tokyo, Japan), connected to an inverted microscope (IX50; Olympus America, Center Valley, PA, USA).

### Surface markers analysis

After ten days of culture in the presence of M-CSF ± Ibrutinib, cells were detached with Accutase, washed by PBS and labeled for 30 minutes at 4°C with anti-CD14-APC, CD90-PE, HLA-DR-V450, CD206-PerCP (Becton Dickinson), anti-CD68-APC (BioLegend, San Diego, CA, USA), anti-CCR7-PE, CD163-PE (BD Pharmingen, San Diego, CA), anti-CD200R-PE (Serotec, Kidlington, UK). After washing with PBS, at least 20,000 cells were acquired on a FACSCantoII cytometer (Becton Dickinson) and analyzed by FlowJo 9.9.6 software (Tree Star, Ashland, OR, USA).

### ELISA

Human PTX3 was evaluated in culture cell-free SN derived from M-CSF and M-CSF + Ibrutinib treated cells after ten days culture, either in basal condition or after 24 hours stimulation with 1 ng/mL LPS. We used a commercially available ELISA kit (Abcam, Cambridge, UK), according to the manufacturer's protocol.

### Phagocytosis assay

After ten days of culture in presence or absence of Ibrutinib, cells were incubated at 37°C with *Escherichia coli* or yeast cell walls bioparticles labelled with pH-sensitive fluorophores, which do not fluorescence outside the cells, but brightly fluorescence in phagosomes (pHrodo Red E.coli BioParticles Conjugate and pHrodo Green Zymosan A BioParticles Conjugate, Life Technologies), according to the manufacturer's instructions. After detaching cells with Accutase (Thermo Electron Corporation, VIC, Australia), the results were evaluated by flow cytometry (FacsCantoII, equipped with a 488 nm laser; Becton Dickinson, San Jose, CA, USA) using an R-phycoerythrin (PE) emission filter (Escherichia coli) or a Fluorescein isothiocyanate (FITC) filter (zymosan). Results were also evaluated by fluorescence microscopy (Zeiss AxioImager 2, equipped with Apotome.2 and Colibri 2 and Camera Zeiss monochrome 702; images were acquired at magnification 100x, numeric aperture 1,47, with Zen 2). In this case, cells were also counterstained with DAPI (ProLong Gold antifade reagent with DAPI, Life Technologies).

### Measurement of oxygen free radicals

The intracellular content of ROS was detected by using CellRox Deep Red reagent (CellRox Deep Red Flow Cytometry Assay Kit, Thermo Fisher Scientific, Waltham, MA, USA), which is specific for detection of superoxide anion. Cells were or not stimulated with PMA and then they were incubated with 500 nM CellRox Deep Red for 45 minutes at 37°C. Fluorescence intensity was measured by flow cytometry (FacsCantoII, equipped with a 633 nm laser using a 660/20BP emission filter) and fluorescence microscopy. In the latter case, cells were also counterstained with DAPI.

### Cell metabolism assays

#### MTT assay

Cells incubated in the presence or absence of 10 μM Ibrutinib were added with MTT [3-(4,5-dimethilthiazol-2-yl)-2,5-diphenyltetrazolium bromide, Sigma-Aldrich]. This assay, based on the cleavage of the yellow tetrazolium salt MTT to form a blue formazan product by mitochondrial succinate dehydrogenase, was meant to evaluate cell metabolism in same scores of fully viable cells (as evaluated by Trypan blue exclusion method [60]).

#### Staining mitochondria

Cells were incubated for 30 minutes at 37°C in serum-free medium containing 200 nM MitoTracker Deep Red FM (Invitrogen). Then they were detached with Accutase, washed in PBS at 4°C and recorded by flow cytometry (laser at 633 nm and 660/20BP emission filter) and fluorescence microscopy. In the latter case, cells were also counterstained with DAPI.

### Statistics

Student's *t*-test, Mann-Whitney *U* test and Kruskall-Wallis analysis of variance (ANOVA) by ranks were used. Bonferroni *post-hoc* analysis was used where specified. Differences were considered significant for *p* values < 0.05.

## SUPPLEMENTARY MATERIALS FIGURES AND TABLES



## Abbreviations

BTK: Bruton's tyrosine kinase
CLL: chronic lymphocytic leukemia
TEC: tyrosine kinase expressed in hepatocellular carcinoma
BCR: B-cell receptor
ITK: interleukin-2-inducible T-cell kinase
EGFR: epidermal growth factor receptor
fMLP: *N*-formylmethionyl-leucyl-phenilalanine
LFA-1: lymphocyte function-associated antigen 1
M-CSF: macrophage colony-stimulating factor
IIF: Ibrutinib-induced fibrocyte-like cells
Mϕ: M-CSF-differentiated macrophages
SAP: serum amyloid P component
PTX3: Pentraxin 3
SN: supernatants
LPS: lipopolysaccharide
ROS: oxygen free radicals
TLR: toll-like receptor
MTT: 3-(4,5-dimethilthiazol-2-yl)-2
