# Supplementary material for: Monocyte-to-macrophage switch reversibly impaired by Ibrutinib
Source: Oncotarget. 2019 Mar 8;10(20):1943–56. doi: 10.18632/oncotarget.26744 (PMC6443008; doi:10.18632/oncotarget.26744)
Supplement: Supplementary file 1 [file oncotarget-10-1943-s001.pdf]

Monocyte-to-macrophage switch reversibly impaired by Ibrutinib

SUPPLEMENTARY MATERIALS

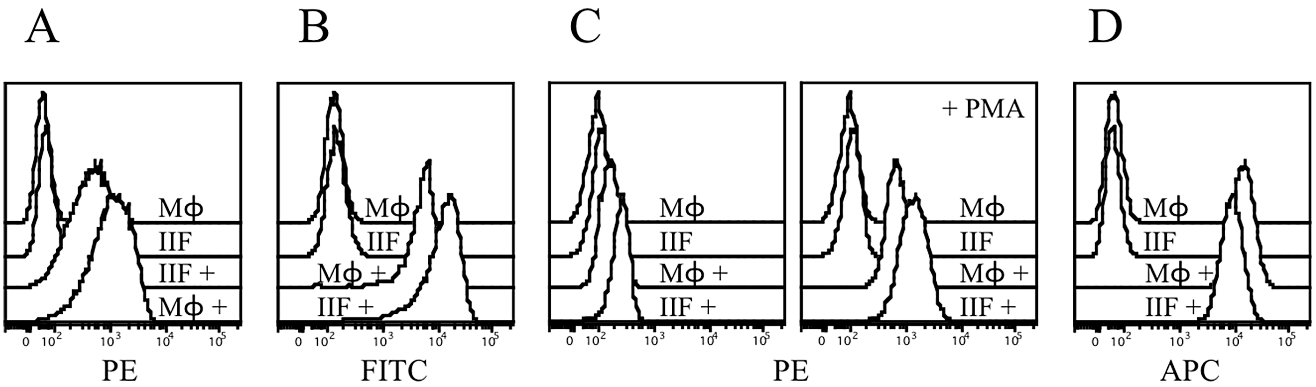

Supplementary Figure 1: Flow cytometry study of macrophages (Mϕ) and Ibrutinib-induced fibrocyte-like cells (IIF). Cells were evaluated for phagocytosis by pHrodo Red E. Coli BioParticles (**A**) and pHrodo Green Zymosan A BioParticles (**B**), ROS production by CellRox Deep Red reagent in the absence or presence of PMA (**C**), mitochondrial mass by MitoTracker Green FM probe (**D**). (+): cells incubated with the specific reagent as specified above. Data are representative of at least 3 experiments.

Supplementary Table 1: Mϕ and IIF surface markers analysis

	Mϕ	IIF
**CD14**	66630 ± 1490	70512 ± 2598
**CD68**	126 ± 15	120 ± 21
**HLA-DR**	18410 ± 510	11770 ± 454*
**CCR7**	negative	negative
**CD206**	3715 ± 1204	3672 ± 1518
**CD163**	470 ± 123	516 ± 112
**CD200R**	1586 ± 510	1641 ± 485

$^{*}p < 0.05$.

Flow cytometry analysis was performed after 10 days of culture. At least 20,000 cells were acquired on a FACSCantoII cytometer (Becton Dickinson) and analyzed by FlowJo 9.9.6 software (Tree Star). Data are expressed as mean ± SD of Mean Fluorescence Intensity of 4 experiments.
